# Proteomic Profiling Reveals Region-Specific Brain Responses During Acclimation to Elevated Water Temperature in Atlantic Salmon (*Salmo salar* L.)

**DOI:** 10.3390/proteomes14030037

**Published:** 2026-07-23

**Authors:** Manojkumar Chandraprakasham, Gianluca Amoroso, Chris G. Carter, Chloe J. English, Lambertus Koster, Richard Wilson, Richard S. Taylor, Omar Mendoza-Porras

**Affiliations:** 1 Institute for Marine & Antarctic Studies, University of Tasmania, Hobart, TAS 7001, Australia; gianluca.amoroso@utas.edu.au (G.A.); chris.carter@utas.edu.au (C.G.C.); omar.mendozaporras@utas.edu.au (O.M.-P.); 2 Agriculture & Food, Commonwealth Scientific & Industrial Research Organisation, Hobart, TAS 7004, Australia; richard.taylor@csiro.au; 3 School of the Environment, The University of Queensland, Brisbane, QLD 4072, Australia; chloe.english@uq.edu.au; 4 Agriculture & Food, Bribie Island Research Centre, Commonwealth Scientific & Industrial Research Organisation, Woorim, QLD 4507, Australia; bert.koster@csiro.au; 5 Central Science Laboratory, Science and Engineering, University of Tasmania, Hobart, TAS 7001, Australia; richard.wilson@utas.edu.au

**Keywords:** Atlantic salmon, brain regions, proteomics, thermal stress, temperature

## Abstract

Background: Increasing summer seawater temperatures pose challenges for Atlantic salmon aquaculture, while brain region-specific responses to elevated temperature remain poorly understood. Methods: Atlantic salmon in the warm treatment (WT) underwent thermal ramping from 15 °C to 19 °C, with mortality observed at the end of week 4 (15.78%). The WT temperature was subsequently reduced to 18 °C in week 5, while the control treatment (CT) was adjusted from 15 °C to 14 °C, and the WT (18 °C) and CT (14 °C) conditions were maintained thereafter. At the end of the trial (week 10), six brain regions, including the cerebellum (CBE), hypothalamus (HYP), medulla oblongata (MED), optic tectum (OPT), pituitary gland (PIT), and telencephalon (TEL), were analysed using data-independent acquisition mass spectrometry-based proteomics. Results: Over 9000 protein groups were identified per brain region, and exploratory differential abundance analysis revealed predominantly region-specific protein abundance changes. Increased SERPINH1 (HSP47) abundance was observed in HYP, PIT, and TEL, suggesting roles in protein-folding and stress regulation. Functional enrichment analyses indicated differential regulation of translation, transcription, energy metabolism, and metabolic pathways across brain regions in the WT group. Conclusions: This study provides a brain region-specific proteomic resource for Atlantic salmon and advances understanding of molecular responses associated with recovery from a temperature reduction (19 °C to 18 °C) and subsequent thermal adjustment at 18 °C.

## 1. Introduction

Atlantic salmon (*Salmo salar* L.) is the most produced marine finfish in global aquaculture [1], with Tasmania (Australia) contributing approximately 86,000 metric tons, valued at $1.3 billion AUD [2,3]. Tasmania experiences generally favourable thermal conditions for Atlantic salmon growth for most of the year [4]. However, during some summers, marine heatwaves have increased sea surface temperatures beyond the optimum growth range (12–18 °C) of Atlantic salmon [5], with temperatures near 19–20 °C leading to reduced feed intake and impaired growth performance [6,7,8]. Elevated temperature has been shown to affect multiple physiological systems in Atlantic salmon, including cellular metabolism [7,9], antioxidant capacity [10], heart morphology and cardiac performance [11,12,13], immune regulation [14,15], reproductive performance [16], flesh colouration [6,17], lipid and fatty acid profiles [18], neural plasticity [19], and osmoregulation [20]. While most studies have focused on peripheral tissues, including liver, muscle, heart, and gill, comparatively little is known about the brain, despite its central role in coordinating stress responses. The fish brain has dual relevance under thermal stress, acting both as the central integrator of neuroendocrine responses [21] and as a metabolically active tissue whose molecular homeostasis can be disrupted by elevated temperatures [19,22,23]. As the control centre, it activates the hypothalamic–pituitary–interrenal (HPI) and brain–sympathetic–chromaffin (BSC) axes, driving cortisol and catecholamine release to mediate metabolic, cardiovascular, and respiratory adjustments essential for adaptation [21,24]. At the same time, the brain’s high energy demand and cellular heterogeneity make it sensitive to temperature-induced changes in membrane composition, neurotransmission, proteostasis, redox balance, and energy metabolism [25,26,27]. A recent study on rainbow trout (*Oncorhynchus mykiss*) showed that acute warming can cause neural failure, suggesting that brain function may limit upper thermal tolerance [28]. In salmonids, acute thermal stress has been shown to induce increased expression of heat shock proteins and apoptotic genes, alongside reduced antioxidant responses [29]. Furthermore, transfer of Atlantic salmon post-smolt to 16–18 °C disrupted stress-axis regulation and suppressed genes associated with neural plasticity in the telencephalon [19]. Acute heat stress responses, including reduced synaptic activity, altered signalling pathways, and endocrine disruption, have been reported in other fish species based on whole-brain transcriptomic analyses [30,31]. However, most existing studies focus on acute heat exposure and whole-brain responses, while the effects of prolonged sub-optimum temperatures remain poorly understood.

Importantly, the fish brain is highly heterogeneous and composed of functionally specialised subregions [32]. For example, the cerebellum regulates motor control and cognition, the hypothalamus–pituitary axis coordinates growth and stress responses, the medulla is involved in autonomic regulation, the optic tectum processes vision, and the telencephalon governs learning and social behaviour [32,33,34,35]. This regional complexity suggests that environmental stressors may drive region-specific responses in the brain. For instance, acute heat stress in Chinese longsnout catfish (*Leiocassis longirostris*) induced distinct transcriptomic responses across brain regions [33], while chronic stress in Atlantic salmon was associated with region-specific changes in serotonergic activity [36]. In recent years, proteomics has been widely applied in aquaculture to investigate the physiological responses of species, including Atlantic salmon, to a range of environmental stressors [7,37,38,39,40]. As proteins are key functional determinants of phenotype, proteomics helps uncover the biological processes and pathways that underpin stress adaptation at the tissue, organ and whole-animal levels [41,42]. In Atlantic salmon, chronic thermal stress alters protein degradation, cytoskeletal organisation, and stress-related pathways in liver tissue [7], and SERPINH1 has been identified as a robust biomarker of thermal stress [37]. In fish brain, proteomic studies in zebrafish (*Danio rerio*) further show that temperature affects energy metabolism, synaptic function, and translational processes [25,26,43]. These findings suggest that thermal stress could induce comparable changes in the proteome of Atlantic salmon brain regions, potentially affecting critical neural and metabolic functions. Owing to the limited understanding of region-specific brain responses and chronic thermal stress in fish, we applied a label-free proteomics approach to explore region-specific proteomic alterations in post-smolt Atlantic salmon exposed to elevated temperature. This study aimed to uncover the underlying molecular mechanisms associated with elevated water temperature in the brain of Atlantic salmon, providing new insights into region-specific proteomic responses.

## 2. Materials and Methods

### 2.1. Experimental Design and Brain Region Sampling

**Figure 1:**
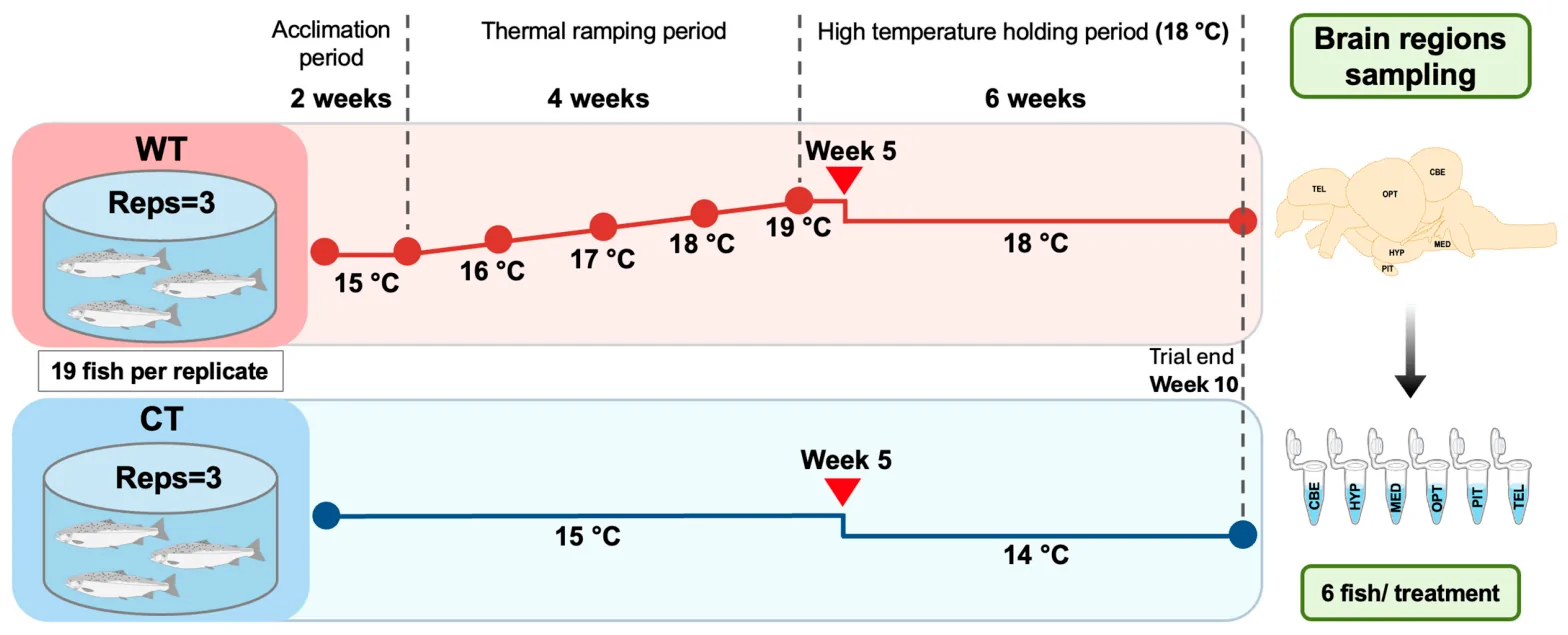
Schematic overview of the experimental design. Atlantic salmon with an initial average weight of 450 g were exposed to a control temperature (CT) and a warm temperature (WT) with *n* = 3 replicate tanks per treatment and *n* = 19 fish per tank. At the end of the experiment, brain region sampling for proteomics was conducted on *n* = 6 fish per treatment (*n* = 2 fish per tank). Arrows (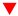) indicate a 1 °C reduction in both treatments in week 5, implemented in response to mortalities in WT while maintaining a 4 °C difference between treatments. Vector element (Eppendorf tube) in the figure was adapted from the NIAID NIH BioArt Source: NIAID Visual & Medical Arts. (10/7/2024). Eppendorf Tube. NIAID NIH BioArt Source. bioart.niaid.nih.gov/bioart/143.com.

The experiment was conducted at the CSIRO Bribie Island Research Centre (Queensland, Australia), and all procedures were approved by the CSIRO Aquatic Animal Ethics Committee (CAA AEC 2022-32). A total of 114 post-smolt Atlantic salmon (~450 g), which had previously been transported as 3.5 g fry from a commercial salmon hatchery in Tasmania, were randomly distributed into six 500 L seawater flow-through tanks at a stocking density of 17 kg·m^−3^ (19 fish tank^−1^). Each tank received aerated seawater (~35.5–36 ppt) at a flow rate of ~5 L min^−1^, with dissolved oxygen (DO) maintained at 90–100% saturation, and a 12 h light:12 h dark photoperiod was maintained throughout the 10-week trial. Fish were fed a 4 mm commercial diet at 1% body weight daily from 9:00 a.m. to 2:30 p.m. using automatic feeders (Arvo-Tec, Evijärvi, Finland). Water temperature (°C), DO (% saturation), salinity, and pH were monitored daily using a handheld meter (YSI Professional Plus, Yellow Springs, OH, USA). Weekly assessments of ammonia, nitrite, and nitrate were conducted using a colourimetric saltwater master test kit (API Fishcare, Chalfont, PA, USA) and were maintained within safe limits. Temperature was applied at the tank level, with three replicate tanks per treatment, which is the experimental unit. Fish were acclimated in all tanks at 15 °C for two weeks. The thermal ramping period began thereafter and was designated as week 1 of the experiment. After acclimation, in three tanks, the temperature was increased gradually by approximately 0.3 °C every second day (equivalent to 1 °C per week) until it reached 19 °C (hereafter Warm Temperature treatment, WT), while the remaining three tanks were maintained at 15 °C (hereafter Control Temperature treatment, CT) (Figure 1). When WT tanks reached 19 °C at the end of week 4, mortality was observed. Cumulative losses in the WT treatment gradually increased towards our animal ethics-approved threshold for this experiment. In accordance with the animal ethics requirement, the water temperature of the WT tanks was therefore reduced from 19 °C to 18 °C in week 5, while the CT tanks were adjusted to 14 °C to maintain the 4 °C temperature difference (Figure 1). No mortalities occurred during the 18 °C exposure period in WT. At the end of the 10-week experimental period, fish were euthanised using 100 mg L^−1^ of AQUI-S (AQUI-S^®^, Lower Hutt, New Zealand). From each tank, five fish were randomly selected, and their weight (g) and fork length (mm) were measured. For proteomic analysis, brain regions from six fish per treatment group were collected, with two fish randomly selected from each of three tanks per treatment. Fish therefore represent analytical units nested within tank-level experimental units. Fish heads were severed posterior to the operculum using a sterile knife, and the skull was cut open dorsally through the frontal bone to expose the brain. The entire brain was gently excised by cutting off the optic and olfactory nerves. Using micro-scissors and a scalpel, six brain regions, including the cerebellum (CBE), hypothalamus (HYP), medulla oblongata (MED), optic tectum (OPT), pituitary gland (PIT), and telencephalon (TEL), were dissected. Each region was placed in a separate labelled tube, immediately snap-frozen on dry ice, and stored at −80 °C for proteomic analysis. Dissecting tools were cleaned and disinfected between each fish using 2% chlorine, followed by 70% ethanol.

### 2.2. Growth Measurements and Statistical Analysis

Growth performance was evaluated using weight gain (WG) and specific growth rate (SGR), calculated as weight gain (%) = [(final weight − initial weight)/initial weight] × 100 and specific growth rate (% d^−1^) = (Ln (final weight) − Ln (initial weight))/experimental days × 100. Final weight, fork length, SGR, WG, and final feed intake are presented as mean ± SD. Growth performance was analysed using tank means (*n* = 3 tanks) as the experimental unit. Individual fish measurements (five fish per tank) were used to calculate tank-level averages for statistical comparisons. Normality and homogeneity of variance were tested using the Shapiro–Wilk and Levene’s tests, respectively. An independent *t*-test was used to compare the differences between CT and WT. Statistical analyses for growth data were conducted using R studio software (version 2025.09.2+418) [44], and results were considered significant at *p* < 0.05. The cumulative mortality rate in the WT group was calculated as cumulative mortality rate (%) = total mortalities/total initial fish × 100.

### 2.3. Sample Preparation for Proteomic Analysis

Brain region samples, CBE, HYP, MED, OPT, PIT, and TEL, were thawed, weighed, and transferred to 2 mL screw cap vials containing a denaturation buffer comprising 7 M urea, 2 M thiourea, 30 mM Tris, and EDTA-free protease inhibitor cocktail (cOmplete™, Roche, Basel, Switzerland). Denaturation buffer was added at a tissue-to-buffer ratio of 1:20–1:40 (*w*/*v*), depending on the weight of the brain regions. Samples were homogenised for 20 s using a bead beater (FastPrep-24™, MP Biomedicals, Irvine, CA, USA). The homogenised samples were subsequently incubated overnight in a thermal shaker (Eppendorf ThermoMixer^®^ C, Hamburg, Germany) at 4 °C and centrifuged at 14,000× *g* for 30 min the following day. The supernatants were then transferred to new 1.5 mL Eppendorf tubes, and the protein concentrations were measured using Pierce 660 nm protein assay (Pierce, Thermo Scientific™, Waltham, MA, USA) with bovine serum albumin (BSA) as a standard. After the protein assay, a 30 μg aliquot of total protein from each sample was sequentially reduced, alkylated, and then digested with trypsin + LysC (Promega, Madison, WI, USA) at an enzyme-to-protein ratio of 1:25, as previously described [45]. The digests were then desalted using C18 ZipTips (Merck Millipore, Burlington, MA, USA), dried in a vacuum concentrator (Genevac miVac Duo, SP Scientific, Waverley, Australia) and reconstituted with 12 µL of HPLC loading buffer (2% acetonitrile and 0.05% TFA in water) prior to HPLC-MS analysis.

### 2.4. Proteomic Analysis

Peptide samples were analysed using a Q-Exactive HF mass spectrometer (Thermo Scientific™, Waltham, MA, USA) coupled with an Ultimate™ 3000 RSLCnano system (Thermo Scientific™, Waltham, MA, USA). Approximately 1 µg of peptides from each sample was injected into the LC system and separated using a 120 min segmented gradient. Peptides were separated using a combination of 20 mm × 75 µm and 250 mm × 75 µm PepMap 100 C18 columns in a trap/elute configuration using a flow rate of 300 nL/min and a column oven held at 45 °C. Data acquisition parameters included a 2.0 kV spray voltage, S-lens RF level of 60, and a heated capillary temperature of 250 °C, all configured in MS Tune software (version 2.11). MS1 spectra were acquired in profile mode at a resolution of 120,000 with an AGC target of 3 × 10^6^, followed by centroid-mode acquisition of MS2 spectra at a resolution of 30,000 and an AGC target of 1 × 10^6^. Data-independent acquisition (DIA-MS) data were generated for all brain region samples, with raw MS files processed using the Spectronaut software (version 18.0) (Biognosys AB, Zurich, Switzerland). A project-specific spectral library was created using a subset of 36 raw files, representing all six brain regions at both temperatures. DIA MS2 spectra were searched against the UniProt Atlantic salmon (*Salmo salar*) reference proteome (ProteomeID: UP001652741) using Pulsar factory settings, generating a library of 61,518 unique peptides mapped to 13,101 protein groups, including 2358 single peptide IDs. For library generation, N-terminal acetylation and methionine oxidation were designated as variable modifications, whereas cysteine carbamidomethylation was designated as a fixed modification. Up to two missed cleavages were permitted, with peptide, protein, and PSM thresholds set at 0.01. This library was then used for protein label-free quantitation (LFQ) and cross-run LFQ intensity normalisation across the samples for each brain region individually according to default Biognosys settings, with the exception that single-hit proteins were excluded. All raw proteomics data generated in the study have been deposited to the PRIDE repository via ProteomeXchange with the identifier PXD070727.

### 2.5. Statistical Analysis of Proteomics Data

The resulting protein abundance matrix containing accession IDs, protein descriptions, gene symbols, and abundance values for the six brain regions (CBE, HYP, MED, OPT, PIT, and TEL) was imported into Perseus for statistical analysis [46]. Data for each brain region were analysed separately. Protein abundance values were first log_2_-transformed, and protein groups identified in fewer than 70% of the samples were excluded from further processing. Missing values were imputed with random values drawn from a normal distribution with a width of 0.3 and a downshift of 1.8. The processed data of each brain region were then used for downstream analysis, including assessment of variance structure using a mixed-effects model, principal component analysis (PCA), differential abundance analysis, and functional enrichment analysis. Temperature treatment was applied at the tank level, with three replicate tanks per treatment group. Accordingly, tanks were considered the experimental units, while individual fish were treated as analytical subsamples for proteomic analysis. To account for the hierarchical experimental design, variance structure was assessed for each region using mixed-effects models prior to PCA and differential abundance analysis, with treatment specified as a fixed effect and tank as a random effect. Principal Component Analysis (PCA) was then performed to investigate variation in protein abundance across samples from each brain region in the R software (version 4.4.2) using the multivariate exploratory data analysis package FactoMineR [47] and visualised with ggplot2 [48]. The mixed-effects models were applied to assess the variance structure of the data but were not used for the primary statistical testing. Differential abundance analysis was therefore performed using fish-level data (*n* = 6 per treatment), and tank was not considered the unit of statistical inference. Differentially abundant proteins (DAPs) between the CT and WT groups were identified using independent *t*-test analysis in Perseus software (version 2.1.5.0). Differential abundant analysis using default settings (FDR < 0.05 and s0 of 0.1) identified DAPs only in the CBE, PIT, HYP, and OPT regions, whereas no proteins met the FDR threshold in the remaining two brain regions (MED and TEL). The limited number of FDR-significant proteins restricted downstream functional interpretation. Therefore, an exploratory differential abundance analysis was performed using a permissive threshold of raw *p* < 0.05 and fold change (FC) ≥ 1.5 or ≤0.67. This approach has previously been used in quantitative proteomics studies to identify putative biologically relevant protein changes when sample size and statistical power are limited [49,50,51,52]. Results obtained from this analysis were considered exploratory and hypothesis-generating. The resulting test statistics were then imported into R to generate volcano plots. Furthermore, an UpSet plot analysis was used to explore the overlap and uniqueness of DAPs across the six regions, using the UpSetR package [53]. Additionally, heatmap analysis of chaperone proteins was performed to assess abundance patterns across samples. Log2-transformed protein abundance values were standardised (z-scores) prior to visualisation. Due to redundancy within chaperone families, a focused heatmap was generated using a subset of proteins showing differential abundance (raw *p* < 0.05 and fold change ≥ 1.5 or ≤0.67) in at least one brain region. Heatmaps were generated in R (version 4.4.2) using the ComplexHeatmap package [54].

### 2.6. Functional Enrichment and Protein–Protein Interaction (PPI) Network Analysis

Following the identification of DAPs, the ClueGO plugin [55] in the Cytoscape software (version 3.10.3) [56] was used for functional enrichment analysis of the subsets of proteins that were either increased in the CT or WT groups. Functional enrichment analyses were performed on exploratory, uncorrected lists of differentially abundant proteins. This analysis focused on identifying enriched Gene Ontology (GO) terms, including biological process (BP), molecular function (MF), and cellular component (CC), as well as Kyoto Encyclopaedia of Genes and Genomes (KEGG) pathways. A two-sided hypergeometric test was used to evaluate statistical significance, and the Benjamini–Hochberg method was applied to control the false discovery rate (FDR). GO and KEGG terms were considered significantly enriched if they had an adjusted *p*-value (Benjamini–Hochberg FDR < 0.05) and were associated with at least two proteins from the input list. Enrichment results were visualised as bubble plots using the ggplot2 package. To further investigate the functional relationships of DAPs for each region, protein–protein interaction (PPI) networks were constructed using the STRING database [57]. The Markov Cluster Algorithm (MCL) was used with default parameters to identify distinct functional protein clusters.

## 3. Results

### 3.1. Growth Metrics and Mortalities

Water temperature did not produce a statistically detectable difference in final weight, specific growth rate (SGR), or weight gain (WG) in the present experiment (Table 1). Final feed intake was not significant between CT and WT groups. The cumulative mortality rate in the WT group over the experimental period was 15.78%, and no mortality was observed in the CT group. In the WT group, mortality was observed in all three tanks, with 3, 4, and 2 deaths recorded in tanks 1, 2, and 3, respectively (Table S1).

### 3.2. Overview of Protein Identification, Principal Component Analysis, and Differentially Abundant Proteins (DAPs)

**Figure 2:**
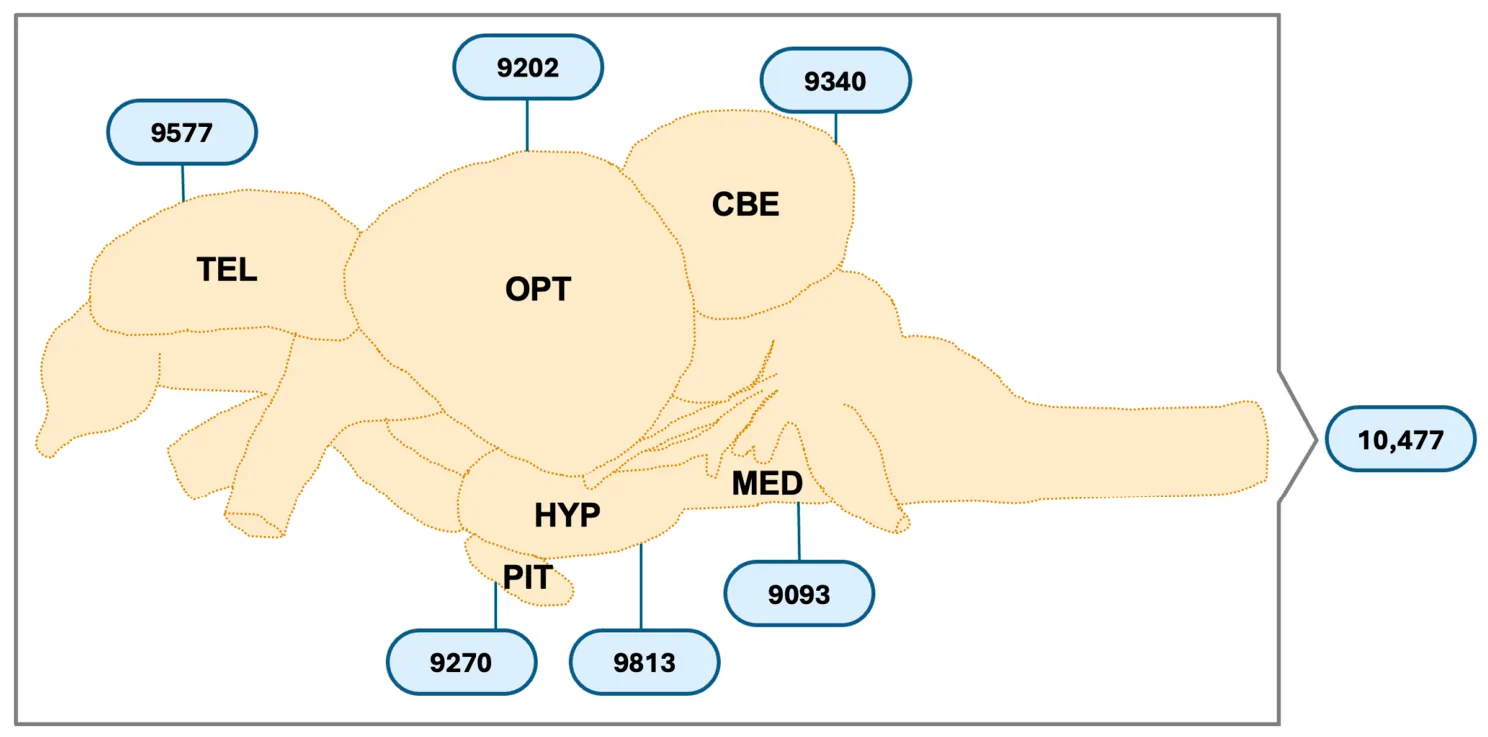
Protein identification across six brain regions in Atlantic salmon. The illustration shows the number of proteins identified in each brain region using DIA-MS-based proteomics with sparse profile quantification. Brain regions include cerebellum (CBE), hypothalamus (HYP), medulla oblongata (MED), optic tectum (OPT), pituitary gland (PIT), and telencephalon (TEL). Across all six regions, a total of 10,447 non-redundant proteins were detected, representing the full set of proteins observed in the brain. A total of 12 samples were analysed for each region, comprising *n* = 6 samples from the CT group (14 °C) and *n* = 6 samples from the WT group (18 °C).).

**Figure 3:**
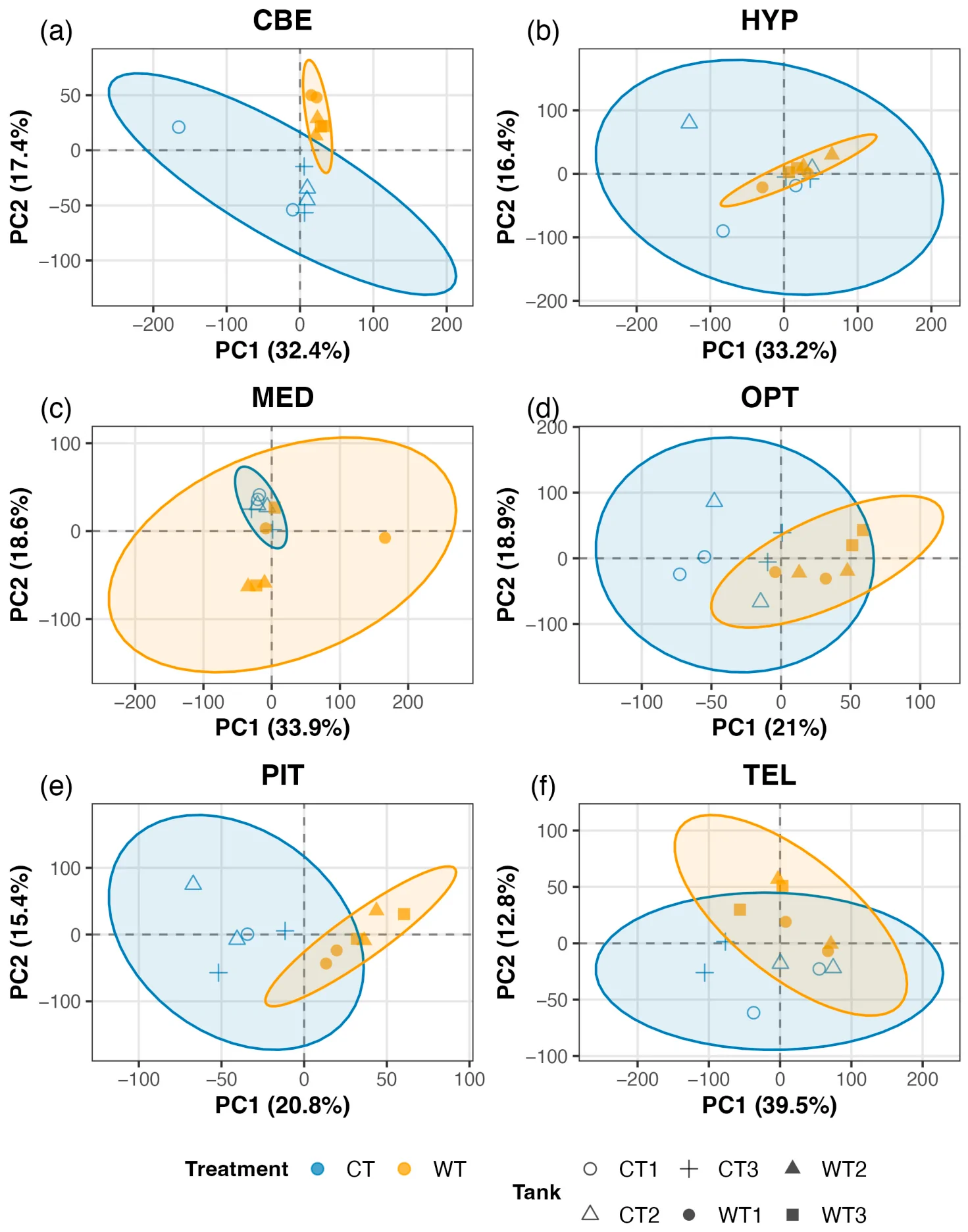
Principal component analysis (PCA) of proteomic profiles in six brain regions of Atlantic salmon. Panels show PCA plots for cerebellum (CBE; (**a**)), hypothalamus (HYP; (**b**)), medulla oblongata (MED; (**c**)), optic tectum (OPT; (**d**)), pituitary gland (PIT; (**e**)), and telencephalon (TEL; (**f**)), illustrating the separation of samples based on protein abundance patterns. Colours indicate treatment groups (blue, CT; orange, WT), and shapes indicate tank identity. The 95% confidence ellipses show within-group variation and the separation between CT and WT samples in PCA space for each brain region.

**Figure 4:**
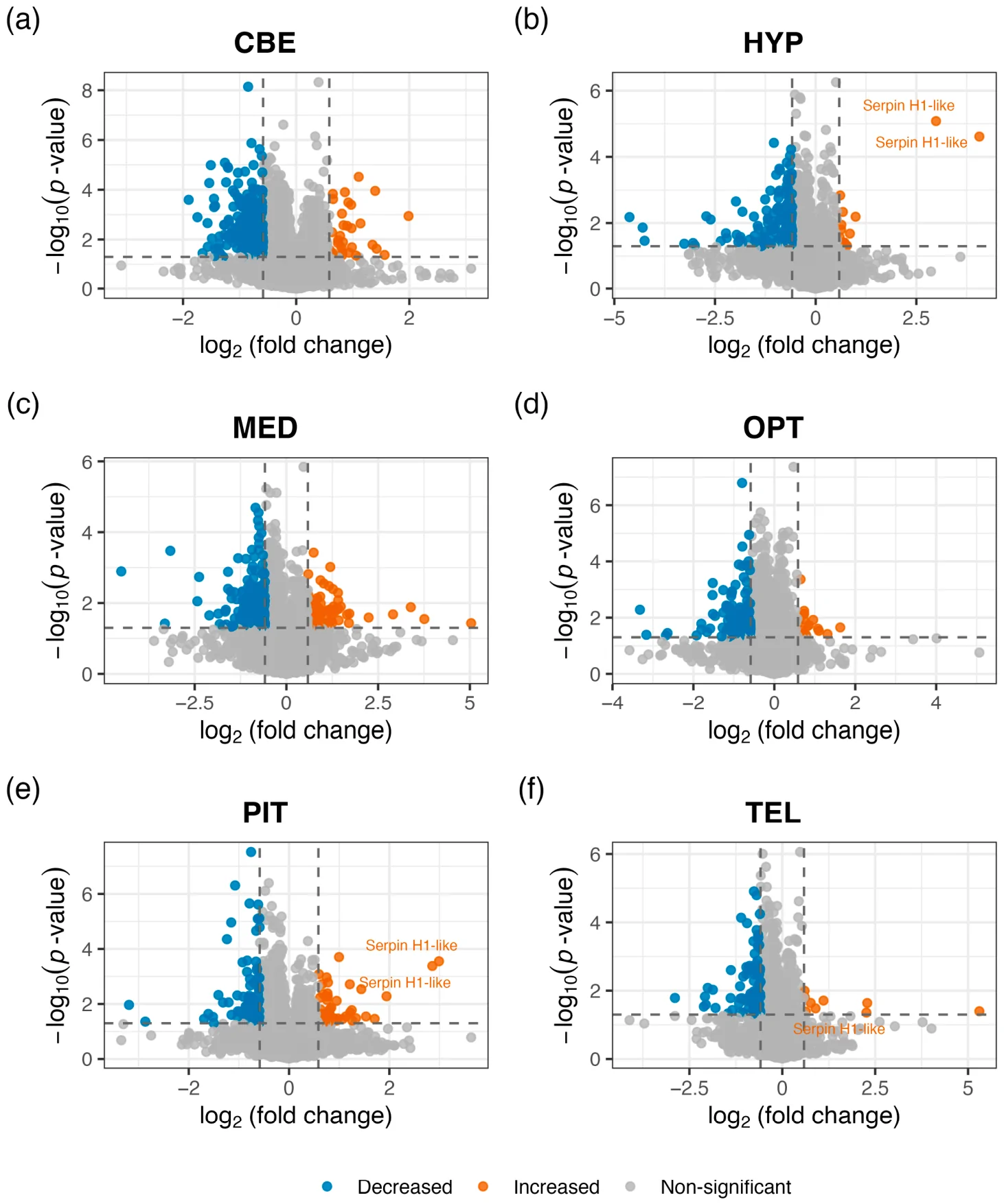
Volcano plots of differentially abundant proteins (DAPs) in the cerebellum (CBE; (**a**)), hypothalamus (HYP; (**b**)), medulla oblongata (MED; (**c**)), optic tectum (OPT; (**d**)), pituitary gland (PIT; (**e**)), and telencephalon (TEL; (**f**)). Red and blue coloured dot points represent the significantly increased (fold change ≥ 1.5 and *p*-value < 0.05) and decreased proteins (fold change ≤ 0.67 and *p*-value < 0.05) in the WT group relative to CT, respectively, and the grey coloured dot points represents the non-significant proteins. The dashed horizontal and vertical lines indicate the significance and fold-change cutoff thresholds, respectively (*p*-value < 0.05; fold change ≥ 1.5 or ≤0.67).

In this study, DIA-MS-based proteomics was used for comprehensive analysis of six different brain regions of Atlantic salmon chronically exposed to control and warm temperatures (14 °C—CT and 18 °C—WT). More than 9000 protein groups (hereafter referred to as proteins) were identified in tissue extracts prepared from the cerebellum (CBE), hypothalamus (HYP), medulla (MED), optic tectum (OPT), pituitary (PIT), and telencephalon (TEL) (Figure 2). The complete set of proteins, together with their intensity values and protein identifiers, is provided in File S1. Mixed-effects model analysis showed that intra-tank variation was greater than inter-tank variation across all brain regions (Figure S1), and treatment effects were larger than tank-level variance (Figure S2). These results suggest limited tank effects and indicate that the observed variation was mainly among individual fish within each tank. PCA revealed varying degrees of separation between the CT and WT groups across the six brain regions (Figure 3). One of the CT samples in the PIT regions was identified as an outlier because it exhibited a markedly distinct protein abundance pattern relative to the remaining samples and disproportionately contributed to the PCA variance. This was further supported by Pearson correlation analysis, which showed lower correlation values (0.68–0.71) for this sample compared with the other PIT samples (File S3). Therefore, the outlier sample was excluded from the PIT PCA and downstream statistical analysis. Moderate separation between the CT and WT groups was observed in the CBE, MED, and PIT, whereas weak separation was observed in the HYP, OPT, and TEL. Using permutation-based multiple correction (FDR < 0.05, *s*0:0.1), we have identified 512 significant proteins in CBE, 98 in PIT, 26 in OPT, 3 in HYP, and none in TEL and MED (File S2). Because the FDR-based approach yielded few or no proteins in some regions, we performed permissive exploratory differential abundance analysis (*p*-value < 0.05; fold change ≥ 1.5 or ≤0.67), which identified 324 exploratory DAPs in CBE (36 increased and 288 decreased), 189 in HYP (17 increased and 172 decreased), 314 in MED (103 increased and 211 decreased), 146 in OPT (16 increased and 130 decreased), 146 in PIT (48 increased and 98 decreased), and 100 in TEL (11 increased and 89 decreased) (Figure 4). In all brain regions, more proteins were decreased in the WT (18 °C) group relative to the CT (14 °C) group. Among the exploratory DAPs across the brain regions, the collagen-specific molecular chaperone SERPINH1 (HSP47) was abundantly increased in WT in the HYP, PIT, and TEL (Figure 4). Other molecular chaperones and co-chaperones related to cell stress, including HSP90A and activator of 90 kDa heat shock protein ATPase homolog 1 (AHSA1), were increased in WT in PIT, while HSP70 was decreased in WT in CBE, HYP, and PIT. Serine/arginine-rich splicing factor 2 (SFRS2) was decreased in WT in CBE, HYP, OPT, PIT, and TEL, but increased in MED. In addition, cytoskeleton-associated proteins were found to be increased in WT in CBE, including tubulin alpha, tubulin-tyrosine ligase, and actin cytoplasmic 1. Further, in WT, fructose-bisphosphate aldolase (ALDOC), a key glycolytic enzyme, was increased in MED and decreased in OPT, PIT, and TEL. The top six DAPs in each region based on fold changes are presented in Table 2, and the complete lists of DAPs in each brain region are provided in File S4 along with the number of quantified and imputed values for each exploratory DAP in both CT and WT groups. A heatmap of all quantified chaperone and co-chaperone proteins (including HSP70 and HSP90 families, HSP40/DnaJ families, SERPINH1, and PDI) showed overall abundance patterns across the six brain regions (File S7; Figure S3). Due to redundancy within protein families (e.g., multiple HSP70, HSP90, and DNAJ protein groups), a focused heatmap based on exploratory differential analysis was generated using selected proteins, including HSP90A, SERPINH1, the DnaJ family, FKBP10, SFRS2, CIRBPB, and AHSA1, which showed region-specific abundance patterns (Figure S4).

### 3.3. Shared and Unique DAPs Across Brain Regions in WT Relative to CT

**Figure 5:**
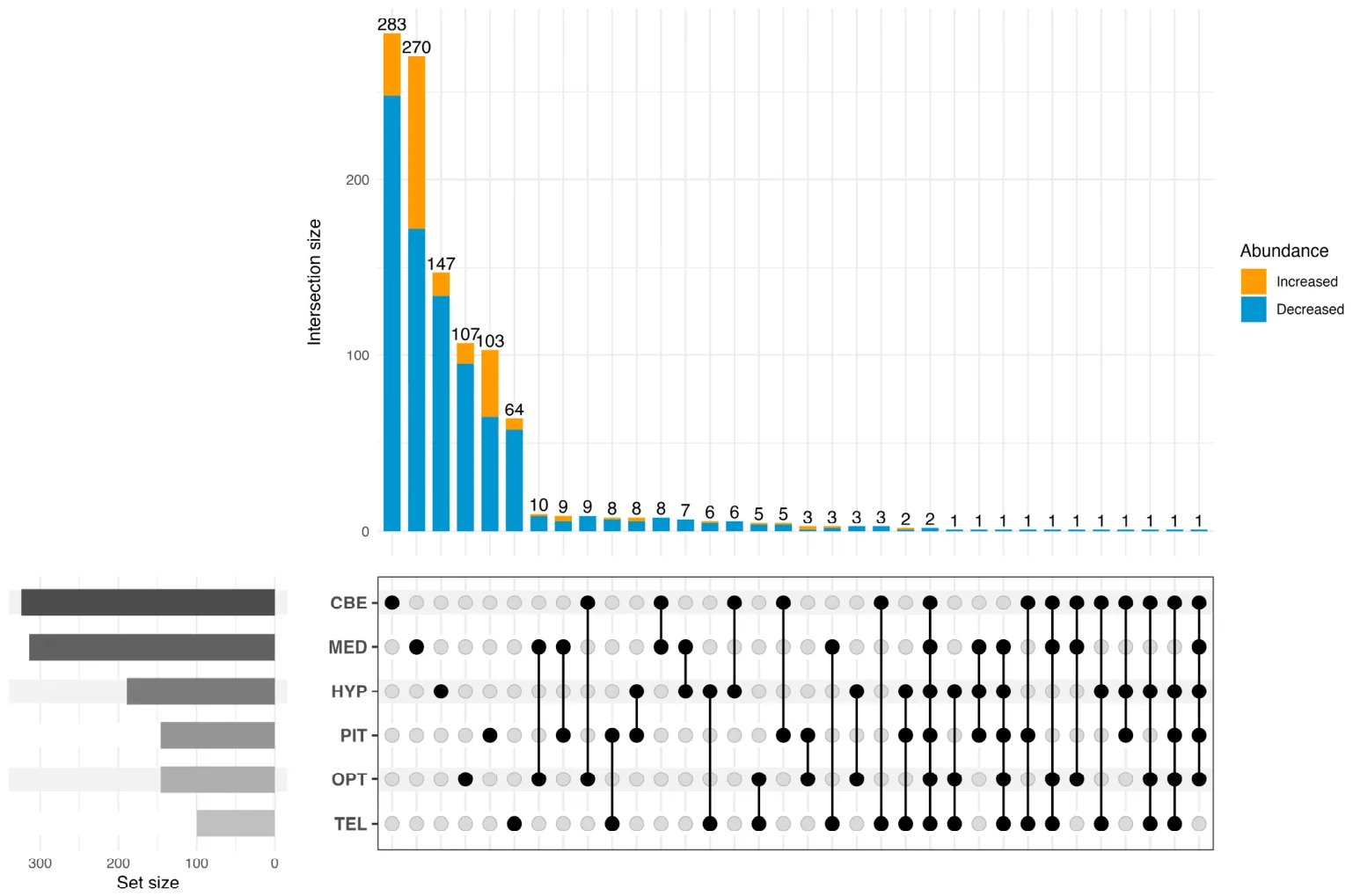
Upset plot analysis of the differentially abundant proteins (DAPs) identified across six distinct brain regions. The upper bar chart represents the number of proteins shared among various combinations of these regions, with blue and orange colours representing significantly decreased and increased proteins, respectively. Below the bar chart, the matrix shows the specific combinations of brain regions contributing to each intersection. The set size shows the total number of DAPs in the cerebellum (CBE), hypothalamus (HYP), medulla oblongata (MED), optic tectum (OPT), pituitary gland (PIT), and telencephalon (TEL).

Shared and unique DAPs between the six regions were then identified using Upset plot analysis (Figure 5). Most of the DAPs were specific to one or more regions, but not all, in the WT group. Only two proteins, adenylosuccinate lyase and RWD domain-containing protein 4 isoform X1, were shared among all the six brain regions.

### 3.4. GO and KEGG Enrichment Analysis of DAPs

#### 3.4.1. Proteins with Increased Abundance in WT Relative to CT

**Figure 6:**
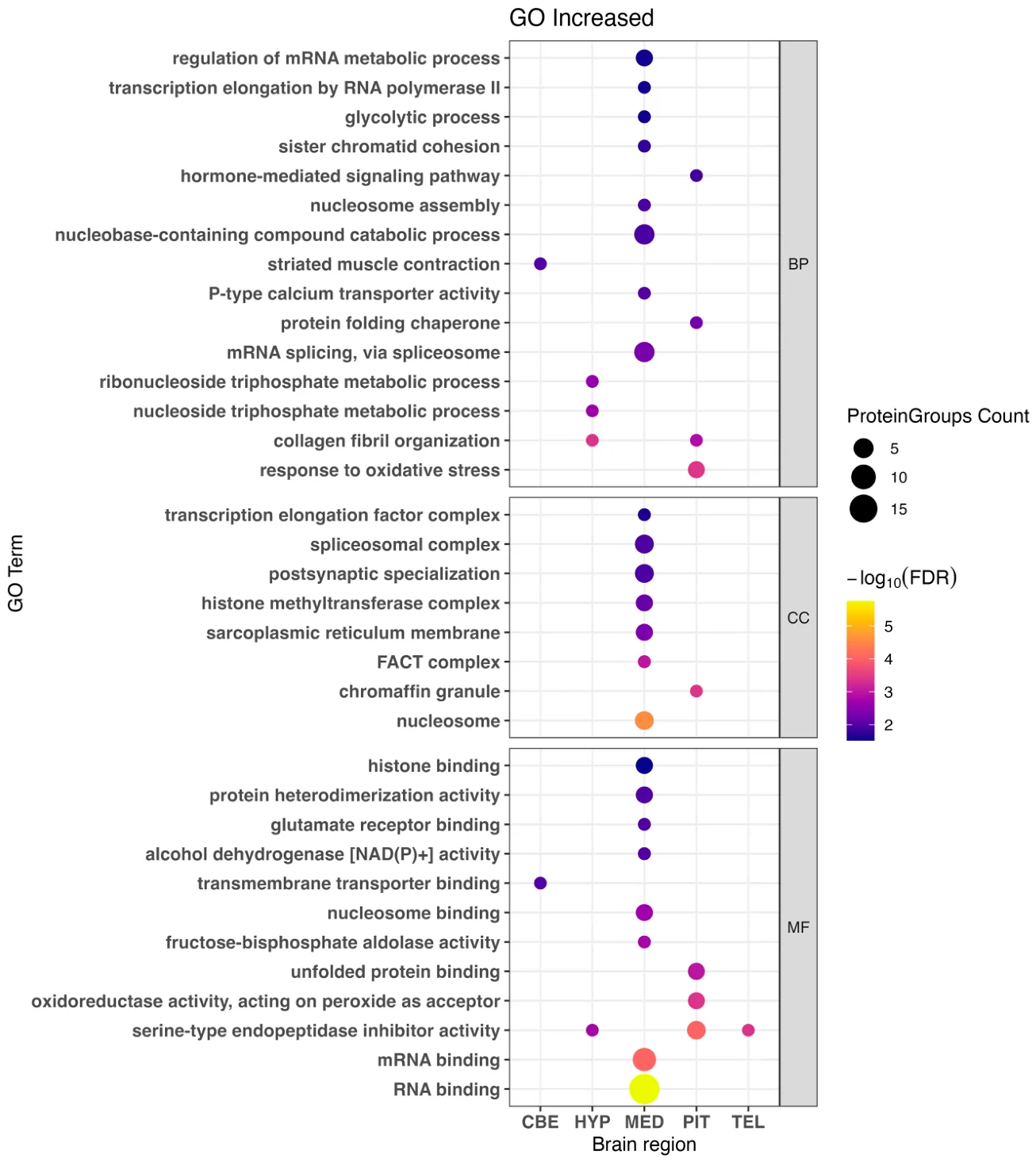
Gene ontology (GO) enrichment analysis of the proteins that were significantly increased at WT relative to CT across the brain regions of Atlantic salmon. Dot plot shows enriched GO terms categorised into biological process (BP), cellular component (CC), and molecular function (MF). The *x*-axis indicates the various brain regions and *y*-axis indicate the top enriched GO terms for abundantly increased proteins. The size of each dot corresponds to the number of proteins associated with each GO term, and the colour represents the statistical significance of enrichment [−log10 (FDR)], with a gradient from dark blue (less significant) to light yellow (more significant).

**Figure 7:**
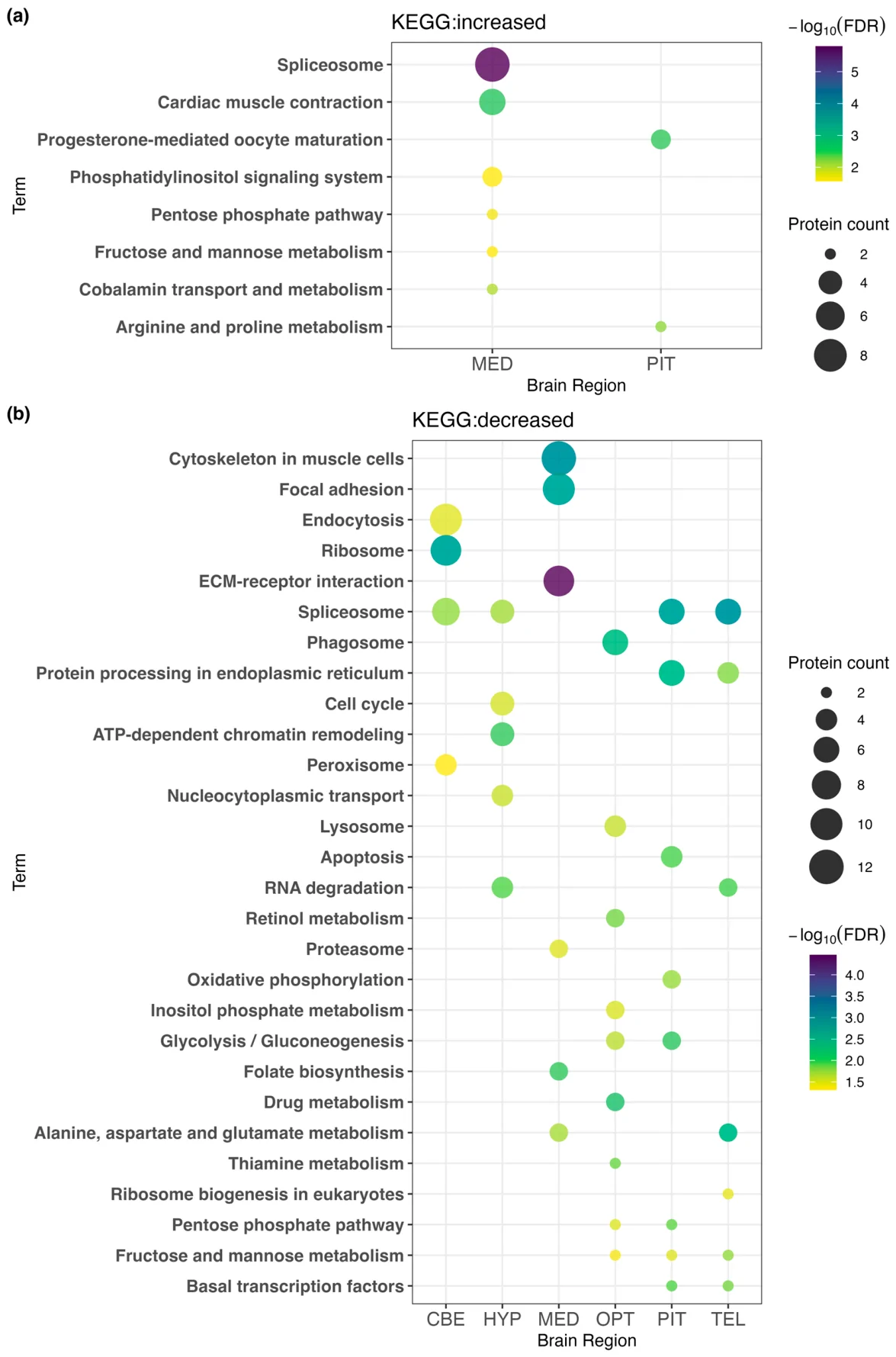
KEGG enrichment analysis of the proteins detected at either increased (**a**) or decreased (**b**) abundance in brain regions of Atlantic salmon exposed to warm temperature (WT). The *x*-axis represents brain regions, while the *y*-axis shows significantly enriched KEGG pathways. Dot size corresponds to the number of differentially abundant proteins associated with each term, and the colour gradient reflects statistical significance based on Benjamini–Hochberg-adjusted *p*-values (FDR < 0.05).

Functional enrichment analysis indicated that the terms protein folding (MF), unfolded protein binding (MF), arginine and proline metabolism (KEGG), and progesterone-mediated oocyte maturation (KEGG) were enriched in PIT in the WT group. In CBE, GO terms related to striated muscle contraction (BP) and transmembrane transporter activity (MF) were significantly enriched. In MED, proteins that were increased abundantly were predominantly associated with mRNA splicing (MF), RNA binding (MF), spliceosomal complex (CC), spliceosome (KEGG), and cardiac muscle contraction (KEGG). No GO or KEGG terms were enriched for the increased proteins of OPT in the WT group. The most significantly enriched GO and KEGG terms for the proteins that were detected at higher abundance at WT in the brain regions are shown in Figure 6 and Figure 7a, respectively, and the complete functional enrichment analysis results are provided in File S5.

#### 3.4.2. Proteins with Decreased Abundance in WT Relative to CT

**Figure 8:**
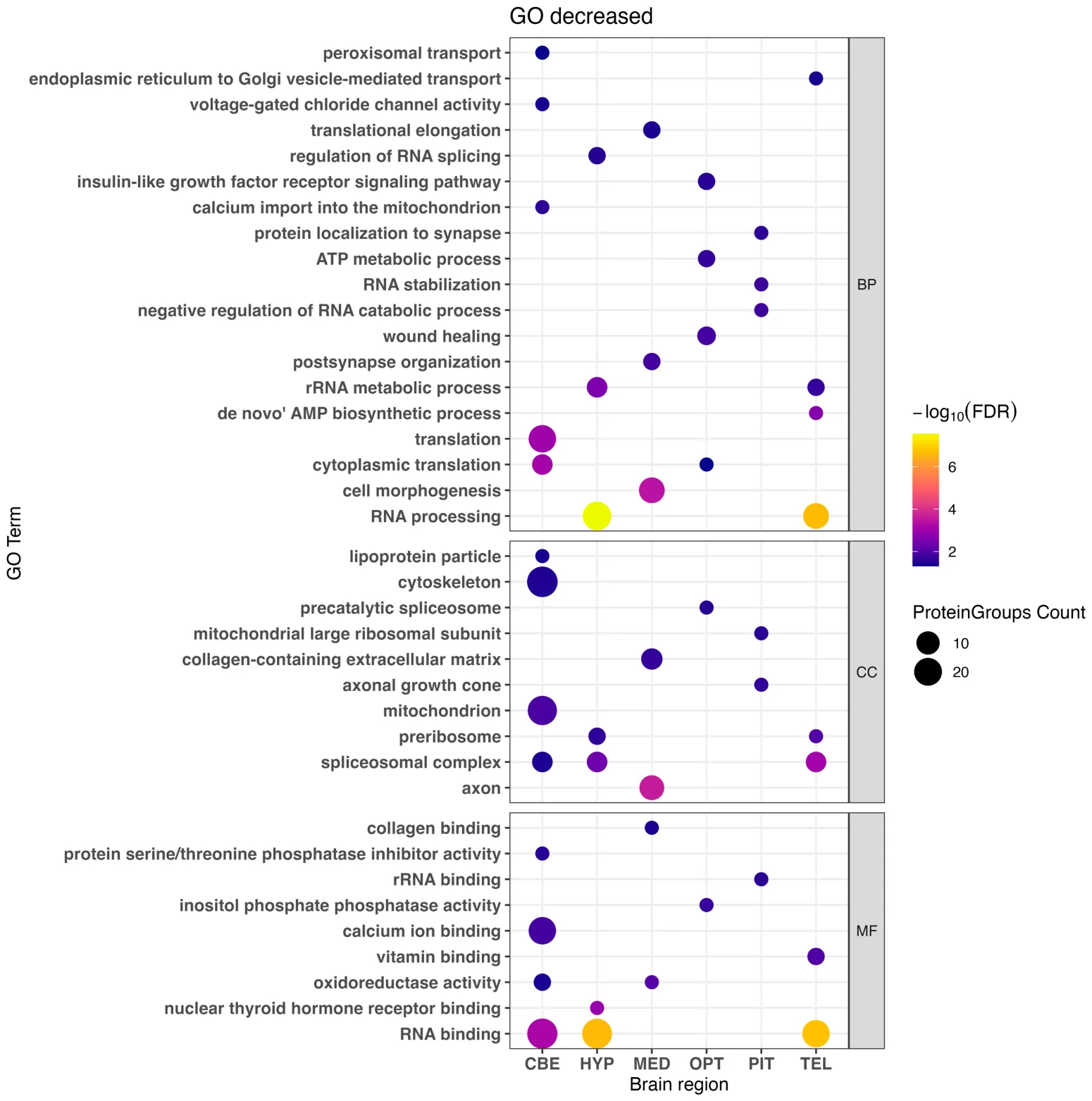
Gene ontology (GO) enrichment analysis of downregulated proteins across brain regions of Atlantic salmon. Dot plot shows enriched GO terms categorised into biological process (BP), cellular component (CC), and molecular function (MF). The *x*-axis indicates the various brain regions and *y*-axis the top enriched GO terms for downregulated proteins. The size of each dot corresponds to the number of proteins associated with each GO term, and the colour represents the statistical significance of enrichment [−log10 (FDR)], with a gradient from dark blue (less significant) to light yellow (more significant).

A greater number of GO terms and KEGG pathways were enriched for the proteins with decreased abundance in all brain regions at WT relative to CT. Most of the GO terms associated with proteins detected at reduced levels at WT were related to the translation and transcription machinery. The identified GO terms included mRNA transport (BP), RNA binding (MF), spliceosomal complex (CC), and RNA processing (BP) in CBE, HYP, and TEL; translation (BP), translation elongation (BP), cytoskeleton (CC), and calcium ion binding (MF) in CBE; glycolytic process (BP) in OPT; and neurogenesis (BP), neuron projection (BP), and amino acid metabolic process (BP) in MED. The decreased proteins showed significant enrichment in KEGG pathways, including spliceosome in CBE, HYP, PIT, and TEL, protein processing in the endoplasmic reticulum in PIT and TEL, RNA degradation in PIT and TEL, phagosome and retinol metabolism in OPT, peroxisome and ribosome in CBE, glycolysis/gluconeogenesis and pentose phosphate pathway in PIT and OPT, and ECM–receptor interaction and focal adhesion in MED. The most significantly enriched GO terms and KEGG pathways for the abundantly decreased proteins in the brain regions are shown in Figure 7b and Figure 8, respectively, and the complete functional enrichment analysis results are provided in File S5.

### 3.5. Protein–Protein Interaction (PPI) Analysis of DAPs

**Figure 9:**
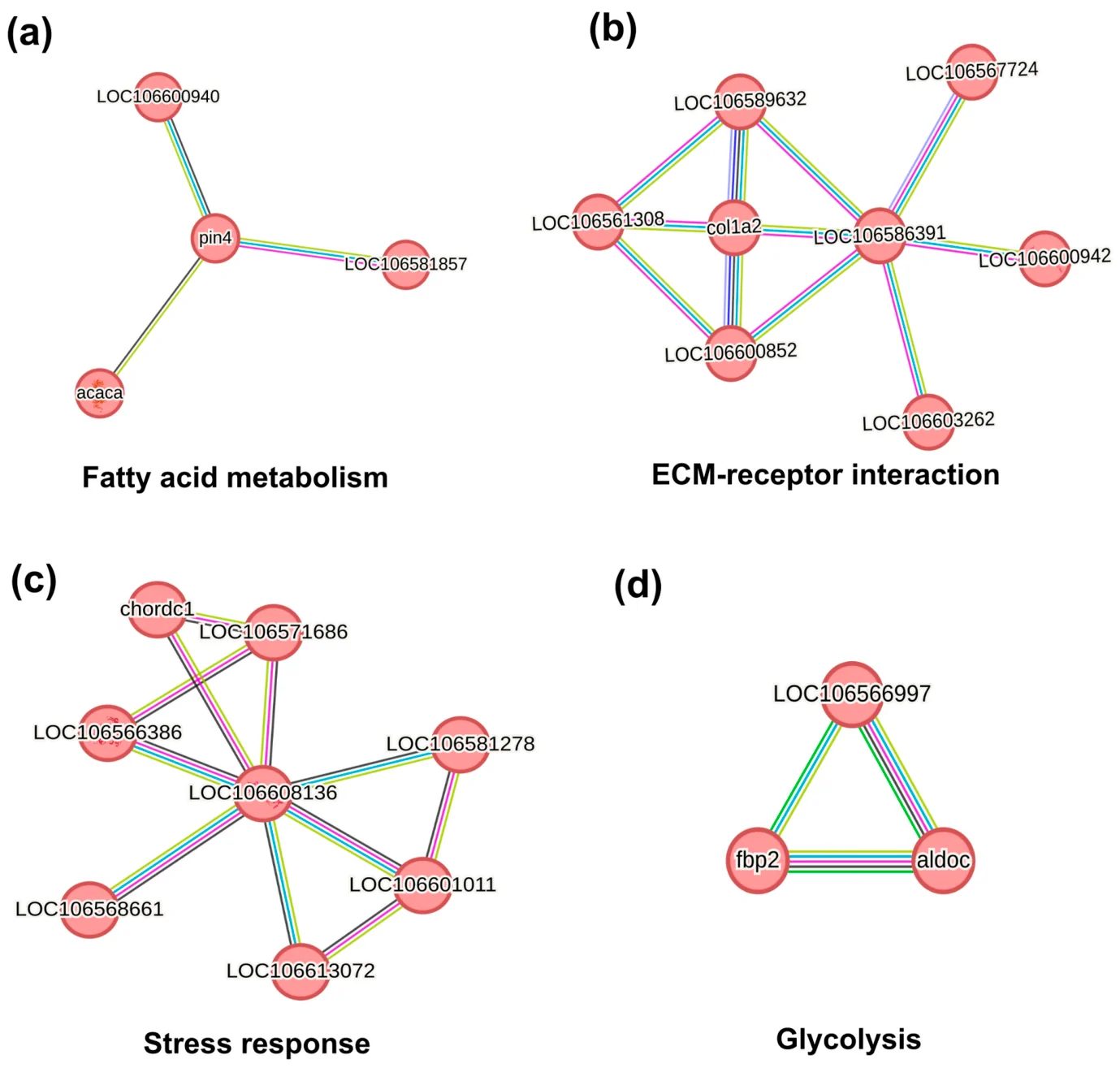
Protein–Protein interaction (PPI) clusters of exploratory differentially abundant proteins (DAPs) in salmon brain regions under elevated water temperature stress. STRING-based MCL clustering analysis of exploratory DAPs revealed distinct functional clusters in various brain regions: (**a**) fatty acid metabolism cluster in the CBE, (**b**) ECM–receptor interaction cluster in the MED, (**c**) stress response and (**d**) glycolysis-related clusters in the PIT. Nodes represent exploratory DAPs, and edges represent protein–protein interactions. Edge colours denote different interaction evidence types: curated databases (light blue), experimentally determined interactions (pink), gene neighbourhood (green), g, gene co-occurrence (dark blue), text mining (light green), co-expression (black), and protein homology (purple). The protein description, FDR value, and associated terms of each cluster are given in File S6.

Next, STRING was used to identify whether subsets of functionally related proteins that were significantly increased in WT relative to CT were involved in protein-interacting networks. The results of MCL analysis identified a number of protein–protein interactions among exploratory DAPs in different brain regions that were consistent with the GO and KEGG enrichment analysis presented above. In CBE, exploratory DAPs formed the PPI network clusters associated with fatty acid metabolism (Figure 9a) and cytoplasmic translation. The PPI network of ECM–receptor interactions (Figure 9b) and focal adhesion was significantly enriched in MED. In PIT, the clusters related to cellular stress and glycolysis were identified (Figure 9c and Figure 9d, respectively).

## 4. Discussion

This study provides a comprehensive analysis of proteomic profiles across the six brain regions of post-smolt Atlantic salmon exposed to elevated water temperature. It is important to note that the WT treatment did not represent a single, stable chronic exposure at 18 °C (refer to Section 2.1). The fish were first exposed to 19 °C, at which mortality occurred in all WT tanks, and were subsequently maintained at 18 °C. Therefore, the WT group represents a survivor cohort that experienced a near-lethal thermal challenge. The observed exploratory proteomic profiles likely reflect a recovery response of the surviving WT population from short-term exposure to 19 °C combined with ongoing physiological adjustments to 18 °C, rather than a uniform chronic thermal acclimation response of the original population. Within this framework, the examined brain regions of surviving WT fish displayed distinct proteomic profiles, with most exploratory DAPs unique to specific brain regions. At the same time, some shared biological processes and pathways across multiple regions suggest a shared response, reflected by changes in proteins, including SERPINH1, and transcription and translation machinery-related proteins. Further, Atlantic salmon represents an evolutionary and taxonomic model for studying vertebrate brain organisation due to its unique history of genome duplication [58]. Salmonids underwent an additional, lineage-specific fourth round of whole-genome duplication (Ss4R), resulting in functional diversification [59]. In this context, the region-specific exploratory proteomic profiles identified here contribute to our understanding of post-stress recovery and thermal stress responses in salmonids and may provide insights into conserved biological processes shared across teleosts.

### 4.1. Elevated Water Temperature Induces Molecular Chaperone-Mediated Responses

Molecular chaperones, including heat shock proteins (HSPs), are highly conserved proteins with a critical role in maintaining cellular homeostasis in fish. As hallmarks of the cellular stress response, the abundance of HSPs is modulated in order to preserve homeostasis by refolding misfolded proteins, preventing protein aggregation, and promoting the degradation of damaged proteins [60,61,62]. In our study, exploratory analysis identified increased SERPINH1 (HSP47) abundance in HYP, PIT, and TEL of the WT group. SERPINH1 is a collagen-specific molecular chaperone [63] that facilitates procollagen folding and stabilisation within the endoplasmic reticulum and supports collagen maturation and extracellular matrix integrity [64,65,66,67]. Unlike many other ER stress-related proteins, SERPINH1 is mainly induced by high temperature [63,68]. The increased expression of SERPINH1 has been reported in previous studies on salmonids’ liver, head kidney, spleen, heart, and gill tissues [37,68,69,70,71], and it has been validated as a biomarker for thermal stress at the transcriptome [67] and proteome levels [37]. In the present study context, increased SERPINH1 abundance in HYP, PIT, and TEL may reflect enhanced collagen-related protein folding and stabilisation during recovery from 19 °C and subsequent maintenance at 18 °C. This regional pattern aligns with previous thermal stress studies reporting stronger protein-folding responses in forebrain regions compared with other brain areas, likely reflecting the important role of these regions in stress regulation [33].

HSP90A and activator of 90 kDa heat shock protein ATPase homolog 1 (AHSA1) were increased in the PIT region of the WT group. Similar to other HSP families, HSP90 binds to ATP and facilitates the refolding of denatured or misfolded proteins [72] and an increased HSP90 expression has been reported in the tissues of salmonids [39,68,71] and other teleost species [31,73]. The PIT-specific induction of HSP90A may be associated with endocrine regulatory activities, as HSP90 is also involved in glucocorticoid receptor maturation and endocrine stress signalling [74,75,76]. In contrast, HSP70 was decreased across the PIT, HYP and CBE regions. This differs from previous studies on salmonids, in which HSP70 levels were increased under both acute and chronic thermal stress [39,40,71,77]. In fish, HSP70 responses are dynamic and vary strongly by species, tissue, stress exposure intensity, stressor type, and sampling time [78,79,80,81]. In the present study, the reduced abundance of HSP70 relative to the control group (14 °C) may reflect attenuation of the initial heat shock response following prior exposure to 19 °C, indicating a transition toward a post-stress recovery. Consistent with this interpretation, HSP70 responses have been shown to be transient, often peaking during early stages of thermal exposure and declining during prolonged exposure or recovery [82,83,84]. Apart from that, the region-specific nature of this response suggests that HSP regulation was not uniform across the brain. Such variation in HSP expression has also been reported in the central nervous system of mammals and birds, indicating that local functional demands can shape stress-responsive pathways [85,86]. Together, these region-specific differential abundance patterns of molecular chaperones suggest a mixed response of partial recovery and adjustment following prior exposure to 19 °C, rather than a uniform heat shock response or stable thermal acclimation state across brain regions.

### 4.2. Region-Specific Adjustments in Cellular and Metabolic Processes

The fish brain is one of the metabolically active organs that relies heavily on glucose metabolism, and environmental temperature fluctuations are known to influence energy metabolism [27]. In the present study, several brain regions, including the pituitary (PIT), optic tectum (OPT), and telencephalon (TEL), showed changes in energy metabolism, with fructose-bisphosphate aldolase C (ALDOC) significantly decreased. ALDOC catalyses a key step in glycolysis [87,88], and its reduced abundance could be indicative of metabolic adjustment following the short-term exposure to 19 °C. In addition to its glycolytic role, ALDOC has been implicated in cytoskeletal organisation, neuronal signalling, and mRNA regulation [88,89,90]. In the brain, glycolytic enzymes are closely associated with neural energy demand, as continuous glucose supply is required to maintain synaptic transmission and cellular maintenance [91,92]. Downregulation of glycolytic enzymes has previously been reported under conditions of metabolic depression, such as anoxia in crucian carp [93]. However, in the present study, the fish maintained at 18 °C showed no mortality, and feed intake and growth remained comparable to CT, potentially indicating that energy balance and physiological performance were maintained. Therefore, the observed changes possibly reflect the region-specific modulation of energy demand and suggest metabolic adjustment processes associated with prolonged 18 °C exposure in the survivor cohort compared to CT (14 °C), rather than an energy limitation or cellular dysfunction.

Consistent with these metabolic changes, multiple components involved in transcription and translation machinery, including ribosomal, spliceosome, and RNA-binding components, were decreased across multiple brain regions (CBE, HYP, PIT, OPT, and TEL). Suppression of protein synthesis-related processes has been reported in salmonids under thermal stress conditions and is commonly associated with reduced cellular activities [67,70]. The observed pattern might indicate a general reduction in protein turnover and RNA processing in fish maintained at 18 °C. So, as transcriptional and translational processes are highly energy-demanding [7], this pattern possibly reflects a regulated downshift in energy-intensive biosynthetic activity during prolonged exposure to elevated temperature (18 °C) following prior acute thermal stress at 19 °C, rather than impaired cellular function. Serine/arginine-rich splicing factor 2 (SFRS2), a key regulator of pre-mRNA splicing and gene expression, was consistently decreased across these regions. This is consistent with previous thermal stress studies on salmonids [69,71]. SFRS2 is involved in constitutive and alternative splicing as well as transcriptional and post-transcriptional regulation [94,95], and its reduced abundance likely suggests modulation of RNA processing and splicing activities. Alternative splicing is a dynamic regulatory mechanism that generates transcript diversity and contributes to functional plasticity in response to environmental conditions, including temperature [94,96,97]. Although temperature-dependent splicing has been reported in fish, studies on Atlantic salmon have primarily focused on disease-related effects [98,99]. The present findings therefore suggest that exposure to temperatures nearing or above the upper thermal limit may also influence spliceosome-associated regulation, highlighting the need for further investigation into post-transcriptional responses in Atlantic salmon during thermal stress and recovery.

In contrast, the medulla (MED) exhibited a distinct pattern characterised by increased levels of ALDOC and proteins related to transcriptional processes and cardiac muscle contraction. Brain regions differ in cellular composition and functional specialisation [100,101], which may contribute to variation in metabolic responses under suboptimum conditions. The medulla is part of brainstem circuits associated with autonomic functions, including respiration and cardiovascular regulation, in vertebrates [34], although direct evidence linking region-specific molecular changes to functional outputs in fish under thermal stress remains limited. Previous studies have shown that thermal stimulation of the fish brainstem can alter ventilation-related activity, indicating that this region is responsive to environmental temperature [102]. Furthermore, brain region-specific transcriptomic studies have demonstrated differences in molecular responses between regions under both optimum [32] and sub-optimum conditions [33,103]. In the present study context, the distinct response observed in the medulla may reflect differential metabolic and cellular adjustment during post-stress recovery and prolonged exposure to 18 °C, potentially linked to its role in autonomic regulation and maintenance of essential brainstem functions under varying temperature conditions.

Proteins related to retinol metabolism, including retinal dehydrogenase 1-like (RDH), retinol-binding protein 1-like (RBP), and UDP-glucuronosyltransferase, were decreased in OPT of the WT group. The optic tectum is the primary visual processing centre in teleost fish, integrating input from retinal ganglion cells to support behaviours such as prey capture, predator avoidance, and navigation [104,105,106]. Retinoic acid (RA), the bioactive metabolite of retinol, is a key regulator of neuronal differentiation and visual system development, and retinol is converted to retinoic acid through a series of enzymatic steps involving retinol-binding proteins and retinol dehydrogenases [107,108,109]. In zebrafish, environmental contaminants reduce RA-related gene expression and disrupt retinal structure and optic tectum function, highlighting the importance of retinol metabolism [110]. Similarly, disruption of RA signalling in avian optic tectum leads to defects in lamination and neuronal migration [107]. Based on our experimental temperature regime, the reduced abundance of retinol metabolism-related proteins can be interpreted as potential modulation of retinoid-related signalling under prolonged exposure to 18 °C compared to 14 °C, which may be associated with altered visual signal processing. However, the functional implications of these temperature-related changes for visual system performance and fish behaviour remain unclear and require further investigation.

In fish, lipids and their constituent fatty acids are essential for energy production and physiological function [18,111]. The brain is a lipid-rich tissue where lipids contribute to membrane structure, myelination, and signal transduction, and disruption of lipid homeostasis has been associated with oxidative stress and impaired neural function in vertebrates [112]. In the cerebellum, efficient lipid metabolism is important for sustaining the energetic and membrane demands of motor coordination, balance, and sensorimotor processing [113]. In the present study, the concurrent reduction of CPT1 and ACACA levels in the CBE region may suggest altered lipid metabolic activity shaped by post-stress recovery processes and temperature-associated modulation at 18 °C. CPT1 is involved in mitochondrial fatty acid transport for β-oxidation, while ACACA regulates fatty acid synthesis and indirectly influences fatty acid oxidation through malonyl-CoA [111,114]. Temperature-associated modulation of lipid metabolism has also been reported in fish. For example, transcriptomic analyses in longsnout catfish showed region-specific changes in lipid-related pathways under temperature variation [33], while Atlantic salmon liver showed downregulation of lipid metabolism-associated proteins at elevated temperature [7]. In zebrafish brain, thermal exposure has been linked to alterations in lipid classes such as gangliosides and ceramides, which may influence membrane properties and neural function [115]. Given the lipid-rich nature of the fish brain and the importance of lipids for membrane fluidity, myelination, and synaptic transmission [18,115], lipidomic analyses would help clarify how temperature influences neuronal cell membrane dynamics, lipid composition, and synaptic function.

## 5. Conclusions and Study Limitations

Overall, the proteomic profiling indicated distinct region-specific responses across the brain under the current experimental thermal regime, with limited overlap between regions. The increased abundance of collagen-specific molecular chaperone SERPINH1 in the forebrain regions (PIT, HYP and TEL) may reflect enhanced requirements for protein folding and extracellular matrix maintenance. Along with forebrain regions, other brain regions also displayed changes in energy metabolism and in transcriptional and translational machinery. Distinct responses observed in the MED and CBE may reflect differences in molecular regulation associated with their roles in motor coordination, sensorimotor integration, and autonomic control. Region-specific proteomics patterns may also arise from variation in neuronal composition, cell type diversity, metabolic demands, and functional specialisation across brain compartments; however, this requires future investigation. Although this study provides important insights into brain region-specific proteomic responses to elevated temperature, several limitations should be considered. Since the present experiment reflects a combined response to post-stress recovery following a near-lethal thermal event and prolonged exposure to 18 °C, the WT group represents a survivor cohort and should not be interpreted as a model of thermal resilience. Future work should maintain a constant elevated temperature to better understand the mechanisms underlying long-term thermal stress. Importantly, this was an exploratory study based on a shotgun DIA proteomics workflow, so the findings represent peptide-level protein inference rather than direct measurement of specific proteoforms. These findings should therefore be considered as preliminary and will require validation in future studies with greater biological and tank-level replication. Interpretation is further constrained by incomplete functional characterisation of salmonid genes, particularly duplicated ohnologs [59,116], which complicates ortholog assignment and pathway mapping for a subset of differentially abundant proteins. In addition, GO and KEGG enrichment analyses were performed using differentially abundant proteins identified based on uncorrected *p*-values, which may have introduced upstream noise into the enrichment results. Further, this study examined only elevated water temperature under controlled conditions; therefore, the responses described here may not fully capture brain proteomic dynamics in sea-cage environments where elevated temperatures often coincide with varying degrees of hypoxia. To address this, we are currently extending this framework using time-series experiments that combine warming with hypoxia. In conclusion, this study provides insights into region-specific molecular changes in the Atlantic salmon brain associated with recovery from a thermal challenge and subsequent exposure to 18 °C. These findings provide a foundation for future brain-specific studies aimed at better understanding how temperature influences brain function in salmonids under changing climatic conditions.

## Figures and Tables

**Table 1:** Growth metrics of post-smolt Atlantic salmon exposed to control (CT) and warm (WT) temperatures.

Growth Metrics	CT (14 °C)	WT (18 °C)	*p*-Value
Initial weight (g)	447 ± 29.3	457 ± 41.5	
Final weight (g)	610 ± 55.6	667 ± 11.2	0.161
Final fork length (mm)	349 ± 7.49	360 ± 1.97	0.628
WG (%)	38.4 ± 20.2	49.9 ± 17.0	0.495
SGR (%/day)	0.38 ± 0.22	0.53 ± 0.19	0.407
Final feed intake (% body weight/day)	0.76 ± 0.04	0.79 ± 0.11	0.563

Values are displayed as mean ± SD of *n* = 3 replicate tanks per treatment, with *n* = 5 fish per tank.

**Table 2:** List of top three abundantly increased and decreased proteins in each brain region of Atlantic salmon based on fold change.

Protein Accession	Protein Description	Region	Fold Change (log2)	*p*-Value	CT (Quantified/Imputed)	WT (Quantified/Imputed)
A0A1S3RSM0	Tubulin–tyrosine ligase (EC 6.3.2.25)	CBE	2.9	0.007	4/2	6/0
A0A1S3R6L5	THO complex subunit 6 homolog	CBE	1.9	0.001	5/1	6/0
A0A1S3L1C8	Coiled-coil domain-containing protein 82-like	CBE	1.9	<0.001	4/2	6/0
A0A1S3L9E6	Intermediate filament protein ON3-like	CBE	−3.3	<0.001	6/0	3/0
A0A1S3M7V4	Iron-sulfur cluster assembly 1 homolog, mitochondrial	CBE	−1.9	0.03	6/0	3/0
A0A1S3LCA8	Prothymosin alpha-like isoform X1	CBE	−1.8	<0.001	6/0	6/0
A0A1S3T1G8	SERPIN H1 (Collagen-binding protein)	HYP	3.8	<0.001	5/1	6/0
A0A1S3NTG6	SERPIN H1 (Collagen-binding protein)	HYP	2.9	<0.001	6/0	6/0
A0A1S3PJR1	Netrin-G2-like isoform X1	HYP	1.7	0.03	3/3	6/0
A0A1S3MBK6	Bifunctional lysine-specific demethylase and histidyl-hydroxylase	HYP	−4.6	0.006	6/0	6/0
A0A1S3RLS8	Double C2-like domain-containing protein	HYP	−4.2	0.01	6/0	6/0
A0A1S3P120	Unconventional myosin-X-like	HYP	−4.0	0.03	6/0	5/1
B5XB68	Uracil phosphoribosyltransferase *	MED	5.0	0.03	6/0	6/0
A0A1S3P2G9	Glutamate receptor	MED	3.7	0.02	6/0	6/0
B5X0T0	Fructose-bisphosphate aldolase	MED	3.3	0.01	6/0	6/0
A0A1S3P472	Microfibrillar-associated protein 2 isoform X1 *	MED	−4.9	0.04	3/3	6/0
A0A1S3MUK8	Ras-associated and pleckstrin homology domains-containing protein 1-like	MED	−4.5	0.001	6/0	6/0
A0A1S3RUW9	D-ribitol-5-phosphate cytidylyltransferase	MED	−3.3	0.03	6/0	6/0
A0A1S3R4U1	Uncharacterised protein LOC106599937	OPT	2.0	0.04	4/2	5/1
A0A1S3NY85	Follistatin-related protein 1	OPT	1.7	0.02	3/3	6/0
A0A1S3SI48	T-complex protein 11-like protein 2	OPT	1.4	0.04	4/2	6/0
A0A1S3SZJ5	Fructose-bisphosphate aldolase	OPT	−3.2	0.03	6/0	4/2
A0A1S3KQC8	Engulfment and cell motility protein 3-like isoform X1	OPT	−3.1	0.02	6/0	3/3
A0A1S3KR99	MAP7 domain-containing protein 2-like	OPT	−3.2	0.004	6/1	5/0
A0A1S3T1G8	SERPIN H1 (Collagen-binding protein)	PIT	3.4	<0.001	5/0	6/0
A0A1S3NTG6	SERPIN H1 (Collagen-binding protein)	PIT	3.3	<0.001	5/0	6/0
A0A1S3MUZ7	Phospholipase A2 inhibitor 31 kDa subunit-like	PIT	3.6	<0.001	4/1	6/0
A0A1S3Q0E8	3-oxo-5alpha-steroid 4-dehydrogenase (NADP(+))	PIT	−2.8	0.02	4/1	6/0
A0A1S3KWZ7	Fibrillin-1-like	PIT	−1.8	0.004	5/0	3/3
A0A1S3LAT1	TBC1 domain family member 20-like	PIT	−1.6	0.03	5/0	4/2
B5X3T1	Cerebellin-1 (Cerebellin-3 precursor) *	TEL	5.2	0.04	4/2	5/1
A0A1S3PAQ3	Acetyl-CoA carboxylase kinase	TEL	2.2	0.02	6/0	6/0
A0A1S3NTG6	SERPINH1 (Collagen-binding protein)	TEL	2.2	0.04	5/1	6/0
A0A1S3NJ03	Glycogenin-1-like	TEL	−2.9	0.01	5/1	4/2
A0A1S3S5G1	SH3 and cysteine-rich domain-containing protein 2-like	TEL	−2.0	0.01	6/0	6/0
A0A1S3LKS7	Tubulin alpha chain	TEL	−2.0	0.02	6/0	6/0

Proteins marked with an asterisk (*) showed large fold changes with marginal *p*-values and should be interpreted cautiously. These values are influenced by partial missingness (imputation) in one group and/or low-abundance measurements across samples in one group.

## Data Availability

The mass spectrometry proteomics data have been deposited to the ProteomeXchange Consortium via the PRIDE partner repository [117] with the dataset identifier PXD070727 and can be accessed using the following reviewer account: username: reviewer_pxd070727@ebi.ac.uk; password: QlHMfOUBCzlO.

## Supplementary Materials

The following supporting information can be downloaded at: https://www.mdpi.com/article/10.3390/proteomes14030037/s1, File S1: Spectronaut output file containing protein group intensities, protein group accession IDs, protein descriptions, and gene symbols for all six brain region samples; File S2: FDR-significant proteins across CBE, PIT, HYP, and OPT brain regions. File S3: Pearson correlation analysis of PIT region samples; File S4: Exploratory differentially abundant proteins list of all the brain regions; File S5: GO and KEGG enrichment analysis results of differentially abundant proteins for all brain regions; File S6: Protein–protein interaction analysis results; File S7: Protein abundance of all quantified chaperones across brain regions samples; Figure S1: (**a**) Inter- and intra-tank variation across six brain regions. (**b**) Variance ratio (intra/inter) across six brain regions; Figure S2: (**a**) Comparison of inter-tank and treatment effects across six brain regions. (**b**) Ratio of inter-tank to treatment effects across six brain regions; Figure S3: Heatmap of region-specific abundance patterns of chaperone proteins across all samples; Figure S4: Heatmap of a subset of significantly different proteins across brain regions in CT and WT groups; Table S1: Tank-level mortality in the warm (WT) and control (CT) group.

## Abbreviations

The following abbreviations are used in this manuscript:

CBE	Cerebellum
HYP	Hypothalamus
MED	Medulla oblongata
OPT	Optic tectum
PIT	Pituitary gland
TEL	Telencephalon
CT	Control
WT	Warm temperature
DIA	Data independent acquisition
GO	Gene ontology
MF	Molecular function
BP	Biological process
CC	Cellular component
KEGG	Kyoto Encyclopaedia of Genes and Genomes
