# Supplementary material for: Proteomic Profiling Reveals Region-Specific Brain Responses During Acclimation to Elevated Water Temperature in Atlantic Salmon (Salmo salar L.)
Source: Proteomes. 2026 Jul 23;14(3):37. doi: 10.3390/proteomes14030037 (PMC13510651; doi:10.3390/proteomes14030037)
Supplement: Supplementary file 1 [file proteomes-14-00037-s001.zip › Table S1.pdf]

Supplementary Table S1. Tank level mortality in the warm (WT) and control (CT) group

Treatment	Tank	Initial fish	Number of mortalities	Mortality %	Fish used for proteomics
WT	1	19	3	15.7	2
WT	2	19	4	21.0	2
WT	3	19	2	10.5	2
CT	1	19	0	0.0	2
CT	2	19	0	0.0	2
CT	3	19	0	0.0	2
